# Socioeconomic and behavioral determinants of non-compliance with physician referrals following community screening for diabetes, hypertension and hyperlipidemia: a mixed-methods study

**DOI:** 10.1038/s41598-023-47168-8

**Published:** 2023-11-23

**Authors:** Sungwon Yoon, Hendra Goh, Jie Kie Phang, Yu Heng Kwan, Lian Leng Low

**Affiliations:** 1 Health Services and Systems Research, Duke-NUS Medical School, Singapore, Singapore; 2 Centre for Population Health Research and Implementation (CPHRI), Singapore Health Services, Singapore, Singapore; 3 Saw Swee Hock School of Public Health, National University of Singapore, Singapore, Singapore; 4 SingHealth Internal Medicine Residency Programme, Singapore, Singapore; 5 Population Health and Integrated Care Office (PHICO), Singapore General Hospital, Singapore, Singapore; 6 Department of Family Medicine and Continuing Care, Singapore General Hospital, Singapore, Singapore; 7 Post-Acute and Continuing Care, Outram Community Hospital, Singapore, Singapore; 8 SingHealth Duke-NUS Family Medicine Academic Clinical Program, Singapore, Singapore

**Keywords:** Endocrinology, Risk factors

## Abstract

Early detection of undiagnosed diabetes, hypertension or hyperlipidemia through screening could reduce healthcare costs resulting from disease complications. To date, despite ample research on the factors linked to the uptake of community health screening programs, little attention has been directed at delayed or incomplete follow-up after positive outcomes are identified in community screening tests. This study aimed to investigate the socioeconomic and behavioral factors that influence non-compliance with recommendations for primary care physician referrals, following community-based screening for diabetes, hypertension and hyperlipidemia. A parallel mixed-methods study was conducted. For quantitative data, we performed multivariable analysis on community-based chronic disease screening data. The qualitative component involved semi-structured interviews with individuals with both non-compliance and compliance with referral recommendations. Thematic data analysis was undertaken using the Theoretical Domains Framework (TDF). The quantitative analysis showed that older age (OR = 0.92, 95%CI [0.89–0.96]), non-Chinese ethnicity (OR = 0.24; 95% CI [0.08–0.44]) and residing in 5-room public/ private housing (OR = 0.40; 95% CI [0.14–0.74]) were associated with lower odds of non-compliance with referral recommendations. Thematic analysis identified multiple behavioral-level determinants acting as enablers or barriers within 7 TDF domains: awareness of health risks after receiving screening results, self-management orientation and behavioral control, fear of formal diagnosis and concerns about healthcare cost, optimistic belief driven by the lack of symptoms, interpersonal relationship and social obligations, aversion to medication, communication at the result collection and sense of uncertainty regarding self-scheduling of appointment. Findings provide valuable implications for the development of interventions aimed at improving adherence to referral recommendation. Future endeavors should include culturally sensitive outreach, evidence-based information dissemination, family-centered education, positive public health messaging, brief counseling during result collection and an opt-out appointment system to enhance follow-up care.

## Introduction

Growing evidence suggests that high fasting plasma glucose, high blood pressure, and elevated low-density lipoproteins, collectively referred to as metabolic syndrome, are modifiable risk factors for increased cardiovascular disease (CVD) events^1–3^. These three metabolic syndromes are the leading causes of mortality and morbidity worldwide, accounting for a substantial proportion of deaths and disability-adjusted life years (DALYs). According to the International Diabetes Federation, diabetes alone was responsible for an estimated 6.7 million deaths in 2021 and played a significant role in complications such as kidney failure, lower limb amputation, and visual impairment^4^. Moreover, a report from the World Health Organization indicated that CVD-related deaths attributed to hypertension and hyperlipidemia accounted for an estimated 17.9 million deaths, positioning it as the leading cause of death worldwide^5^. These disease burdens underscore the critical need for early diagnosis to address their shared underlying roots and mitigate the cumulative health risks they pose. As a result, it is crucial to implement effective prevention strategies to reduce the burden of these diseases.

Against this backdrop, many jurisdictions introduced community-based screenings in an effort to prevent disease progression and slow debilitating health outcomes^11^. While facility-based screenings can conveniently leverage existing resources, the substantial patient demand for curative services and the geographical placement of screening sites may serve as deterrents to participation especially among community-dwelling residents^12^. On the other hand, community-based screenings not only foster early disease detection to enable timely interventions but may also promote health equity by reaching underserved populations who may face barriers to accessing traditional healthcare facilities^13,14^. Beyond their clinical benefits, community health screening programs offer valuable opportunities for health education and empowerment, encouraging individuals to take an active role in their well-being^15^. By engaging with communities directly, these screenings facilitate a collaborative approach to healthcare, strengthening the social fabric and contributing to improved health outcomes on a broader scale.

Despite the positive effects outlined in community health screening programs, an issue that warrants attention is the suboptimal follow-up that often ensues. For example, in US and Netherlands, compliance rates for referral following a chronic disease screening in primary care were 63% and 86% respectively^16^. In Singapore where this study was conducted, one in four who were screened for either hypertension, hyperlipidemia or hyperglycemia and received a referral recommendation did not return for a doctor’s follow-up^17^. Failing to adhere to recommended follow-up care and treatment plans significantly increases the risk of disease progression and complications associated with chronic conditions. Low engagement in the recommended healthcare regimen may also result in poorly managed blood pressure, cholesterol levels, and blood sugar, thereby exacerbating the susceptibility to cardiovascular events, organ damage, and other severe health repercussions^18–20^. These findings stressed that while community-based initiatives are effective in identifying health concerns at an early stage, subsequent steps in ensuring comprehensive care and treatment for those detected with conditions may fall short, signaling an urgent need for targeted interventions to improve the adherence rates to follow-up care.

In the literature, extensive research has elucidated the factors influencing the uptake of community screening programs^21–23^. Barriers such as limited access to healthcare facilities, lack of awareness about available services, and financial constraints can impede follow-up effort^24,25^. However, an underexplored aspect exists within the realm of follow-up processes subsequent to these screenings. While past studies have investigated the challenges of referrals, their primary focus has often been on referrals from primary care settings to specialist clinics^16,26,27^. This lack of research underscores the need for a better understanding of the factors influencing post-screening behaviors, particularly in cases where ‘abnormal’ screening outcomes are observed. Undertaking community-based health screening requires substantial investment. If the targeted individuals fail to derive benefits from screenings, the investment in healthcare resources remains suboptimal^28^. Furthermore, poor follow-up can result in unnecessary disease progression and complications, which have a profound impact on an individual’s quality of life^29^. Hence, understanding the determinants of non-compliance is crucial for augmenting the effectiveness of community health screening programs.

The escalating prevalence of diabetes, hypertension, and hyperlipidemia on a global scale has transformed these conditions into pressing public health concerns^30–32^. As these chronic diseases place a significant burden on healthcare systems and contribute to a substantial portion of preventable morbidity and mortality, it is imperative to delve into the determinants of non-compliance and compliance with referral recommendations. Therefore, this study aimed to investigate the socioeconomic and behavioral factors that influence non-compliance with recommendations for primary care physician referrals following community-based screening for diabetes, hypertension and hyperlipidemia.

## Methods

### Setting

This study was conducted in Singapore, a multi-ethnic city-state in Southeast Asia. In Singapore, one in three deaths is attributed to diabetes, hypertension and high total cholesterol^33^. To mitigate the burden of chronic diseases, the government has stepped up efforts to conduct routine chronic disease screening, namely Screen for Life’, for hypertension, hyperlipidemia or diabetes mellitus in the community^34^. Individuals at risk of developing hypertension or hyperlipidemia were given a letter containing screening results in person at the community center or via postal mail and requested that they make an appointment with a primary care physician for further checkup. To encourage them to comply with referral recommendations, the SingHealth cluster implemented an additional measure; nurse practitioners reached out to these individuals, checked referral compliance and explained the potential risks associated with delayed appointments.

### Study design

The study employed a mixed methods approach in which qualitative and quantitative data were collected and analyzed in parallel^35^. The quantitative data assessed socioeconomic factors and certain limited behavioral factors, while the qualitative data was obtained though semi-structured interviews with a subset of the quantitative data participants, aiming to elicit in-depth information about behavioral determinants influencing decisions on non-compliance/compliance. In the discussion section of the study, narrative integration was conducted to synthesize the quantitative and qualitative data, allowing for a more holistic understanding of the research findings. For qualitative data, we used the Theoretical Domains Framework (TDF)^36^. TDF was chosen because it provides sufficient breadth to analyze a wide range of cognitive, social, affective and environmental influences on behaviors (i.e., non-compliance). The TDF comprises 14 validated domains which include (1) knowledge, (2) skills, (3) social/professional role and identity, (4) beliefs about capabilities, (5) optimism, (6) beliefs about consequences, (7) reinforcement, (8) intentions, (9) goals, (10) memory, attention and decision processes, (11) environmental context and resources, (12) social influences, (13) emotion and (14) behavioral regulation.

### Sampling and recruitment

#### Quantitative component

SingHealth, the largest public health cluster in Singapore, administers the national health screening program. One of the study team members overseeing the screening program (LL) facilitated the acquisition of screening dataset spanning from 2016 to 2019. During this period, a total of 1,625 screening cases were observed. We screened the cases according to the following eligibility criteria: (1) an abnormal screening outcome for diabetes, high blood pressure or high blood cholesterol as recorded by a physician in charge of community screening; (2) previously undiagnosed (i.e., not told by a doctor that they have the disease); (3) received a primary care physician referral recommendation; and (4) received follow-up calls by a nurse for up to 6 months.

We collected a set of independent variables for our study. Socioeconomic variables included age, gender, ethnicity, marital status, educational attainment, monthly household income, employment status, residential housing type (as a proxy for socioeconomic status) and whether individuals have a personal family doctor. Behavioral data included smoking status, engagement in physical activity. We also collected data on whether immediate family members formally diagnosed with diabetes, hypertension or hyperlipidemia. The selection of these variables was guided by existing literature. Previous research has indicated that factors such as income, education, and place of residence^37^ are associated with participation in screening tests. Another study found that age, marital status, income level, education, socioeconomic status, smoking and engagement in exercise can influence the uptake of screenings^38^. To categorize average monthly income, we utilized our dataset’s 25^th^ percentile (below $2,000), median range ($4,000-$5,900) and 75^th^ percentile ($6,000 and above) as cutoffs. This categorization aligns with local studies, which have also used thresholds of < SGD$2,000 to define low monthly income and ≥ SGD$6,000 to define high monthly income^39,40^. For smoking status, we adopted categorization of current smoker and ex-smoker or nonsmoker, as employed in previous research^41^. Our categorization for housing type was adapted from prior research^42–44^.

#### Qualitative component

The qualitative component utilized the same cohort as quantitative component to approach eligible participants by telephone. We employed purposive sampling based on compliance status and age to ensure a range of experiences relevant to our research aim*.* We recruited both compliant and non-compliant individuals to gain insight into how various behavioral factors influenced their behavior. This approach allowed us to better understand the specific factors enabling or hindering individual’s decisions regarding compliance. In-depth interviews with individuals were conducted. Written informed consent was obtained prior to the interviews. A semi-structured interview guide was developed. Topics included experience regarding the collection of screening results and physician follow-up recommendations that were relayed by on-site staff, reasons for compliance or non-compliance with the recommendation and experience of nurse follow-up calls (Supplementary material 1). Interviews were conducted by an interviewer trained in qualitative research methods and lasted 30–60 min. The interviews were audio-recorded and transcribed verbatim. This study was approved by the SingHealth Centralized Institutional Review Board (CIRB: 2019–2229).

### Data analysis

#### Quantitative component

Descriptive summary statistics were presented as counts and percentages were used to describe the different sociodemographic composition between the compliant and non-compliant group. Pearson’s Chi-squared test of independence and Student’s t-test were employed to determine if specific sociodemographic factors differ between two groups for categorical and continuous variables. We performed bivariable and multivariable logistic regression models to compute the crude odds ratio and adjusted odds ratio respectively to examine the association between compliance status and socioeconomic characteristics. Variables in the multivariable logistic regression model were chosen based on their Variance Inflation Factor (VIF) values, with those having a VIF score of ≤ 5 being designated as independent variables to assess multicollinearity. To improve model fit, we performed a stepwise backward elimination procedure, with a p-value more than or equal to 0.1 as the significance level for variable removal. Statistical analyses were performed using IBM SPSS Statistics (Version 27). A p-value of less than 0.05 was considered statistically significant.

#### Qualitative component

We adopted both inductive and deductive approaches in data analysis. Inductive thematic analysis was performed involving immersion in the data, coding, repeated sorting, and comparison. Each transcript was coded line by line to create code components. Each component was compared with other components to ensure that they were mutually exclusive. Following iterative comparisons of components, they were grouped into categories and subthemes, which were then continually refined and classified while accounting for deviations. Categories and subthemes were subsequently mapped to the components of the TDF to systematically identify factors influencing compliance. All transcripts were independently coded by two coders (SY, HG). Discrepancies were resolved through an iterative consensus process. We used NVivo 12 for data management and coding. All methods were performed in accordance with relevant guidelines and regulations^45^.

## Results

### Characteristics of participants

#### Quantitative component

During the data collection period, 1,625 individuals completed the screening. Out of these, 528 individuals met the inclusion criteria resulting in the final sample for analysis (Supplementary material 2). The mean age was 60 years old. Around 60% were female, 89% were Chinese and 78% were married. Half of the individuals were in the workforce, and 62% completed secondary school or a higher level of education. A small proportion (7%) were current smokers while 71% reported routinely participating in physical activities. In terms of compliance with referral recommendations, nearly 70% of individuals were non-compliant (Table 1). Of note, only age was significantly different between the two groups (p < 0.05).

**Table 1:** Participant characteristics (quantitative, n = 528).

	Total	Non-compliant	Compliant	*p*-value
Sample size^a^	528 (100.00)	360 (68.18)	168 (31.82)	
Age (in years)^b^	61 (10.6)	62 (10.5)	58 (10.3)	< 0.001
Gender^a^				0.36
Female	312 (100.00)	218 (69.87)	94 (30.13)	
Male	216 (100.00)	142 (65.74)	74 (34.26)	
Ethnicity^a^				0.06
Chinese	472 (100.00)	315 (66.74)	157 (33.26)	
Non-Chinese	56 (100.00)	45 (80.36)	11 (19.64)	
Marital Status^a^				0.08
Single	69 (100.00)	42 (60.87)	27 (39.13)	
Married	416 (100.00)	283 (68.03)	133 (31.97)	
Divorced/Separated/Widowed	43 (100.00)	35 (81.40)	8 (18.60)	
Highest Education Level^a^				0.44
No formal education/ Primary	197 (100.00)	140 (71.07)	57 (28.93)	
Secondary	190 (100.00)	129 (67.89)	61 (32.11)	
Post-secondary	141 (100.00)	91 (64.54)	50 (35.46)	
Employment status^a^				0.21
Not working	271 (100.00)	192 (70.85)	79 (29.15)	
Working	257 (100.00)	168 (65.37)	89 (34.63)	
Housing type^a^				0.42
1/2-room flats	88 (100.00)	60 (68.18)	28 (31.82)	
3/4-room flats	315 (100.00)	209 (66.35)	106 (33.65)	
Others^a^	125 (100.00)	91 (72.80)	34 (27.20)	
Average monthly income^a^				0.55
Below $2000	130 (100.00)	87 (66.92)	43 (33.08)	
$2000-$5999	62 (100.00)	39 (62.90)	23 (37.10)	
$6000 and above	158 (100.00)	106 (67.09)	52 (32.91)	
NIL	178 (100.00)	128 (71.91)	50 (28.09)	
Currently seeing a doctor^a^				0.81
No	493 (100.00)	335 (67.95)	158 (32.05)	
Yes	35 (100.00)	25 (71.43)	10 (28.57)	
Smoking status^a^				0.23
No/ Ex-smoker	487 (100.00)	336 (68.99)	151 (31.01)	
Yes	41 (100.00)	24 (58.54)	17 (41.46)	
Engage in physical activities^a^				0.08
No	148 (100.00)	92 (62.16)	56 (37.84)	
Yes	380 (100.00)	268 (70.53)	112 (29.47)	
Immediate family members ever diagnosed with chronic diseases^a^				0.69
No	237 (100.00)	159 (67.09)	78 (32.91)	
Yes	291 (100.00)	201 (69.07)	90 (30.93)	

^a^Presented in terms of number (percentage).

^b^Presented in terms of mean (standard deviation).

^c^Includes 5-room/ Executive condominium / Landed property/Others (Condominium/Private flat).

#### Qualitative component

We approached a total of 38 individuals, and 26 agreed to participate in the interviews. Reasons for the decline included being busy or lacking interest in participation. Data saturation was reached at 23 interviews, with no new categories and sub-themes emerging under or outside the TDF domains relevant to non-compliance/compliance with referral recommendations during subsequent interviews. To achieve point of information redundancy, three more interviews were conducted beyond data saturation. The age of participants spanned from 41 to 74, with a mean age of 56 years. The majority were Chinese (84%) and married (80%) (Table 2).

**Table 2:** Participant characteristics (qualitative, n = 26).

	Total	Non-compliant	Compliant
Number of respondents	26 (100.00)	17 (65.38)	9 (34.62)
Age (in years)			
Mean age (standard deviation)	56 (9.0)	54 (7.7)	60 (10.0)
Age range	41–74	43–67	41–74
Gender^a^			
Male	18 (100.00)	13 (72.22)	5 (27.78)
Female	8 (100.00)	4 (50.00)	4 (50.00)
Ethnicity^a^			
Chinese	22 (100.00)	14 (63.64)	8 (36.36)
Malay	2 (100.00)	2 (100.00)	0 (0.00)
Indian	2 (100.00)	1 (50.00)	1 (50.00)
Education^a^			
None/ Primary	9 (100.00)	5 (55.56)	4 (44.44)
Secondary	4 (100.00)	3 (75.00)	1 (25.00)
Post-secondary	13 (100.00)	9 (69.23)	4 (30.77)
Marital status^a^			
Single/Never married	3 (100.00)	2 (66.67)	1 (33.33)
Married	21 (100.00)	15 (71.43)	6 (28.57)
Divorced/ Widowed	2 (100.00)	0 (0.00)	2 (100.00)
Employment status^a^			
Working	15 (100.00)	10 (66.67)	5 (33.33)
Retired/ semi-retired	9 (100.00)	5 (55.56)	4 (44.44)
Unemployed	2 (100.00)	2 (100.00)	0 (0.00)

^a^Presented in terms of number (percentage).

### Factors associated with non-compliance/compliance

#### Quantitative component

A summary of socioeconomic and behavioral factors associated with non-compliance with physician referral recommendation is found in Table 3. At the bivariable level, older people (OR=0.96; 95% CI [0.95-0.98]) had lower odds of being non-compliant. Furthermore, non-Chinese (OR=0.49; 95% CI [0.25-0.97]) had lower odds of being non-compliant compared to Chinese. At the multivariable level, analyses revealed that after controlling for all covariates in the model, age (OR = 0.92, 95%CI [0.89–0.96]) and ethnicity (OR=0.24; 95% CI [0.08-0.44]) remained negatively associated with non-compliance amongst the participants. Meanwhile, housing type was also found to have a negative association with non-compliance, with individuals in 5-room public or private housing (OR=0.40; 95% CI [0.14-0.74]) had lower odds of being non-compliant compared to those residing in 1- to 2-room public housing (Table 3).

**Table 3:** Socioeconomic and behavioral factors associated with non-compliance with physician referral recommendation (n = 528).

	Unadjusted			Adjusted^b^			
	Odd ratio	[95% CI]	*p*-value	Odd ratio	[95% CI]		*p*-value
Age	0.96	[0.95–0.98]	0.00	0.92	[0.89–0.96]		0.00
Gender (%)							
Female	*Ref*						
Male	1.21	[0.83–1.75]	0.32				
Ethnicity (%)							
Chinese	*Ref*			*Ref*			
Non-Chinese	0.49	[0.25–0.97]	0.04	0.24	[0.08–0.44]		0.00
Marital status (%)							
Single	*Ref*						
Married	0.73	[0.43–1.24]	0.24				
Divorced/Separated/Widowed	0.36	[0.14–0.88]	0.03				
Highest education level (%)							
No formal education/ Primary	*Ref*						
Secondary	1.16	[0.75–1.79]	0.50				
Post-secondary	1.35	[0.85–2.14]	0.20				
Employment status (%)							
Not working	*Ref*						
Working	1.29	[0.89–1.86]	0.18				
Housing type (%)							
1/2-room flats	*Ref*			*Ref*			
3/4-room flats	1.09	[0.66–1.80]	0.75	0.54	[0.26–1.88]		0.16
Others^a^	0.80	[0.44–1.45]	0.47	0.40	[0.14–0.74]		0.02
Currently seeing a doctor (%)							
No	*Ref*						
Yes	0.85	[0.40–1.81]	0.67				
Smoking status (%)							
No/ Ex-smoker	*Ref*						
Yes	1.58	[0.82–3.02]	0.17				
Engage in physical activities (%)							
No	*Ref*			*Ref*			
Yes	0.69	[0.46–1.02]	0.07	0.68	[0.45–1.03]		0.06
Immediate family members ever diagnosed with chronic diseases (%)							
No	*Ref*						
Yes	0.91	[0.63–1.32]	0.63				

^a^Include 5-room/ Executive condominium/ Landed property/Others (Condominium/Private flat).

^b^Backward elimination was performed to improve model fit, X^2^ (6, N = 528) = 35.50, *p* < .001, AIC = 639.16.

#### Qualitative component

**Figure 1:**
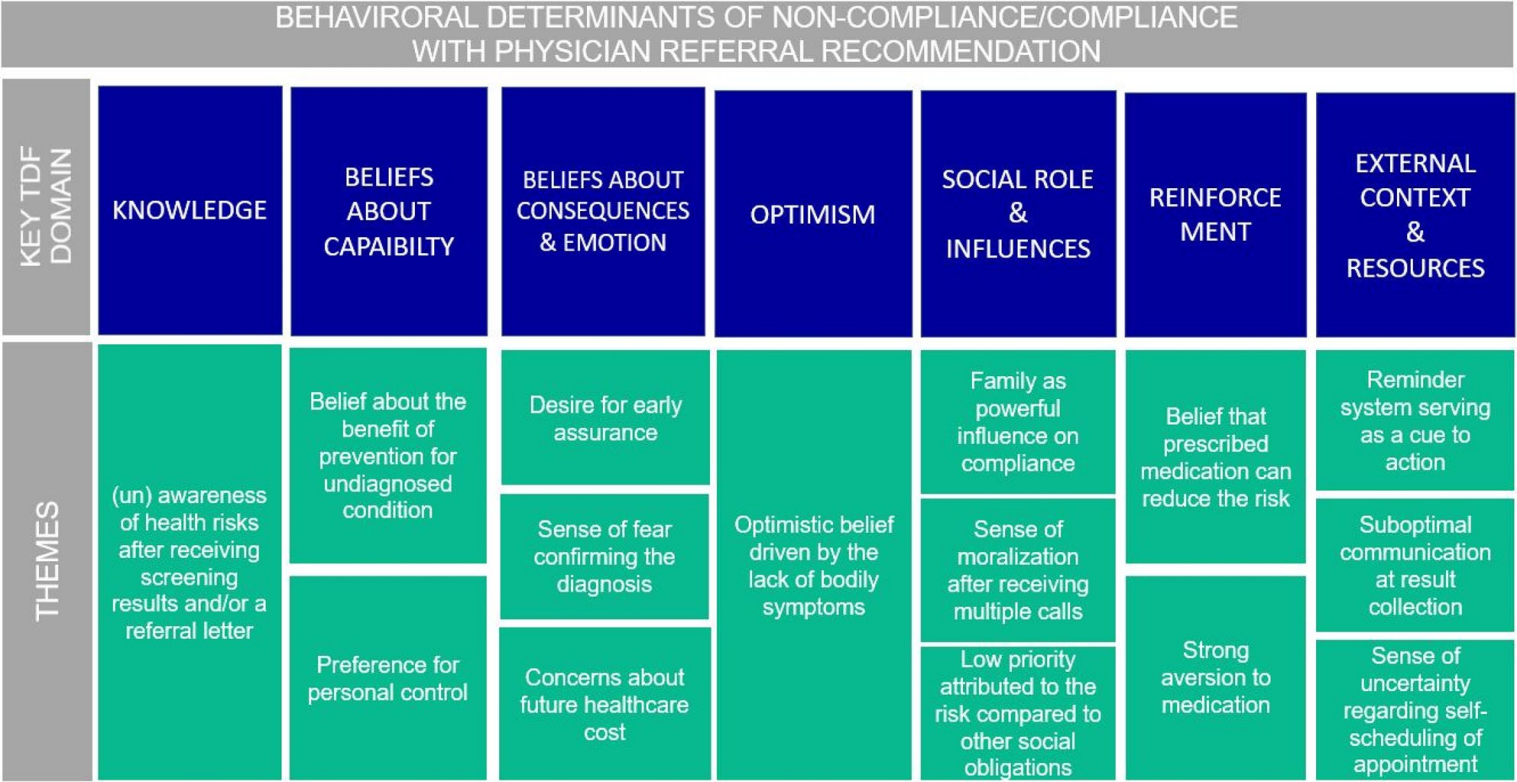
Key TDF domains and behavioral determinants of non-compliance/compliance with referral recommendation.

Our thematic analysis revealed several important behavioral factors acting as enablers or barriers to compliance under various TDF domains. By and large, out of 14 domains of TDF, seven domains were relevant to understanding behavioral determinants of non-compliance/compliance (Fig. 1).

### Knowledge

One of the issues surrounding compliance was the awareness of health risks associated with undiagnosed conditions. Individuals who complied with the physician referral recommendation understood their personal risk and the need for follow-up. On the other hand, those who declined to comply with the recommendation generally lacked awareness of their health risks. Some participants offered a fatalistic view that justified their inaction or a passive approach to health.

*“Because I found out [from screening] that my blood pressure is high, I should go and get more checkup… Since I knew that, I wanted to see what the doctor would recommend.” (#2, compliant)*

*“I know how I am doing. It is not about my cholesterol or the food I eat; it is something to do with my genetics. So, no matter what I try, things won’t really change.” (#P11, non-compliant)*

### Belief about capabilities

There was a consistent pattern within the data regarding beliefs of capabilities acting as either an enabler or barrier to compliance behaviors. Believing that undiagnosed diabetes, hypertension or hyperlipidemia could be prevented significantly influenced the willingness to visit a physician. This perceived behavioral control appeared to foster a sense of autonomy and prompted compliance. Conversely, for individuals with non-compliance, compensatory strategies such as perceptions of personal control reduced the perceived necessity of visiting a physician.

*“I felt I could do something about this [high cholesterol]. So that made me want to see the doctor. But I told the doctor I wanted to manage it myself. I want to see if my condition gets better in a few months or a year without medical treatment.” (#P11, non-compliant)*

*“I didn’t see [a doctor] because I wanted to handle things on my own, I started making changes to my lifestyle. I am trying to exercise more and watch what I eat. I am sure my cholesterol has gotten better since the last time [screening].” (#P24, non-compliant)*

### Belief about consequences and emotions

Regardless of the compliance status, participants commonly appraised whether there would be positive or negative consequences of visiting a physician. Although anxiety about a formal diagnosis prevailed for both groups, a “peace of mind” or desire for early assurance encouraged individuals with compliance to make an appointment with a physician. They believed that a physician visit would be important for their future health outcomes. In contrast, the sense of fear of confirming the diagnosis and its associated future healthcare cost was a powerful barrier among those with non-compliance. Anticipating that they may have other chronic conditions notably reduced the likelihood of a physician visit.

*“After seeing the [screening] results about my blood pressure, I got worried about having a stroke. I thought it would be better to see a doctor just to be safe.” (#P17, compliant)*

*“I always have this worry that if I visit the clinic and get thorough testing, I am afraid I can find out lot of other health issues, you know, then it feels like there is no end to it.” (#P25, non-compliant)*

### Optimism

Optimism, which could be unrealistic, played a key role in hindering non-compliance. This optimism was grounded in their belief that illness is always symptomatic. The lack of bodily symptoms influenced their perceptions that they were not at risk and thus did not feel the need for further actions.

*“It wasn’t like very high, ridiculously high or high to worry about. It’s just that blood pressure is on the high side, nothing more. If it becomes extremely serious, I will definitely go immediately.” (#P24, non-compliant)*

*“I think the key point is I am doing well, you see. I feel great, so why should I spend the time and money to go through another health check?” (#P3, non-compliant)*

### Social roles and influences

The TDF describes social influences as the interpersonal processes that can change the way a person feels or behaves. Social support and expectations were found to be both enablers and barriers. Individuals with compliance described that family members were a powerful influence on their decision to make an appointment with the physician. Receiving multiple calls from a nurse also engendered a sense of moralization to drive these individuals to do the “right thing.” On the other hand, for individuals with non-compliance, social roles became the common factor affecting the decision to delay compliance with the referral recommendation. They described that their own healthcare needs were secondary to work commitments or childcare demands.

“*The nurse called me several times telling me to go for a follow-up. I thought it was not nice to turn them down repeatedly. My daughter had been observing the situation and said why didn’t you go? That’s when I decided to schedule an appointment.” (#P7, compliant)*

*“I’m aware that my blood pressure might be high. But I haven’t been able to see the doctor because I’m busy, I have to take care of my son. I will plan to see the doctor when I have some free time in the future.” (#P23, non-compliant)*

### Reinforcement

The perceived belief that prescribed medications could reduce one’s risk and prevent further deterioration acted as a reinforcement to motivate individuals to comply with the recommendation. Conversely, a strong aversion to medications was a key barrier reinforcing their decision not to make a visit to a primary care clinic.

*“I understand that this thing can be managed by taking necessary medications and staying active. That’s why I quickly visited the doctor at the polyclinic and got some prescriptions” (#P20, compliant)*

*“[I chose not to go] because taking a pill wouldn’t solve the root issue. The medication only controls symptoms, it doesn’t tackle the real problem. If you rely on medication too much, maybe you will have kidney problems down the line.” (#P11, non-compliant)*

### External context and resources

The system of reminder calls by the nurse to follow up with primary care physicians was an enabler for individuals with compliance. In contrast, miscommunication with community screening staff at the point of result collection served as a main barrier to compliance. Additionally, non-compliant participants reported that self-scheduling for an appointment was a challenge for them.

*“I was really glad that they [the nurse] got in touch with me; it felt like they cared about me and my health. That’s what pushed me to go see a doctor.” (#P4, compliant)*

*“On the day that results were given out, there was no mention of needing to consult a doctor, so I thought everything was fine. The staff mentioned my [lab] results were okay.” (#P11, non-compliant)*

*“If they had scheduled an appointment, I would have definitely done the follow up. Since they left the choice of whether to or where to go up to me, I found it difficult to make a decision.” (#P11, non-compliant)*

## Discussion

Adherence to referral recommendations subsequent to community health screening is vital in order to ensure early detection and well-timed management for persons with undiagnosed diabetes, high blood pressure or high blood cholesterol. While the quantitative data revealed some socioeconomic and behavioral determinants, interviews provided behavioral nuances and contexts leading to decisions on non-compliance/compliance. These two sets of results combined to offer a fuller understanding of why a segment of individuals with undiagnosed conditions opted against follow-up despite the need to consult a medical professional to understand their health risks and manage them in a timely manner.

Our quantitative findings suggest that some socioeconomic factors such as younger age, Chinese ethnicity and residing in less affordable housing were associated with non-compliance. Although no straightforward comparison is possible due to sparse literature on this topic, it has been generally recognized that people of older age, being married, and higher education are more likely to participate in preventive health screening than their counterparts^21,46,47^. In addition, overseas studies corroborate that younger age and types of neighborhoods were key determinants of non-adherence to follow-up appointments with both primary and specialist care. For instance, Cristel et al. observed a reduced likelihood of compliance with specialist care referrals among patients residing in socioeconomically disadvantaged urban areas and those aged between 18 and 44 years^26^. In a separate study, Adrian et al. noted a tendency among patients aged 18 to 39 years to forgo general practitioner consultations for confirming screening results^48^. Our finding also revealed an intriguing observation that non-Chinese individuals (i.e., Malays and Indians) exhibited a greater tendency toward compliance in comparison to the Chinese population. This finding stands in stark contrast with the finding from a previous study, which indicated that Malays were less likely to attend health screening compared with the Chinese^23^. The difference in outcomes can be explained by the timing of the two studies. Our study was conducted after the implementation of strategic initiatives designed to engage and support the Malay and Indian community^49–51^. As a result, it is reasonable to assume that the higher compliance rates among non-Chinese individuals in our study could potentially be linked to the successful approach that considers ethnic disparities when addressing health issues among different ethnic groups. These findings, together with ours, indicate the need for tailored outreach efforts for particular segments of the population, such as younger age groups and individuals with low socioeconomic status. In addition, given Singapore’s diverse ethnic makeup, it is imperative to continue developing tailored outreach and education initiatives that incorporate culturally sensitive materials and multilingual resources, to further enhance accessibility and health literacy among different ethnic groups^52^. Lastly, effective policies should be formulated to facilitate collaboration and partnerships with local community organizations, recognizing their pivotal role in reaching out to residents and potentially serving as key facilitators in ensuring ongoing follow-up after health screenings^53,54^.

The quantitative analysis reveals that health behaviors such as current smoking and physical activity did not show a significant association with non-compliance decisions. This contrasts with a previous study that found positive effects of health behaviors on the acceptance of health screenings.^56^ This finding may be explained by the fact that the majority of our participants, irrespective of their compliance status, were non- or ex-smokers (92%) and engaged in regular physical activity (72%). Our interview data further indicates that many non-compliant individuals declined referral recommendations because they believed their self-management efforts were sufficient and strongly preferred personal control (*belief about capabilities*). Consequently, they did not perceive the need for a physician follow-up. However, it is worth noting that maintaining motivation to sustain healthy behaviors and revert early stages of a condition can be a challenge, as supported by existing literature^57,58^. Future intervention may benefit from providing evidence-based information on disease progression through various channels (e.g., national health agencies, expert testimonials) to enhance awareness of the importance of physician referrals.

Within the TDF framework, we identified seven domains relevant to non-compliance/compliance behaviors. One key determinant was *social roles and influences,* which acted as both a barrier and an enabler. While the support received from family members could encourage individuals, social obligations and relationships often hindered them from prioritizing their health over other competing social demands. This finding resonates with existing literature that interpersonal dynamics and role expectations significantly influence health-seeking behaviors^59–61^. Although our quantitative analysis did not show a direct influence of family members’ chronic diseases on non-compliance behavior, we noted that 55% (201 out of 360) of family members of non-compliant individuals had previously been diagnosed with chronic diseases. Given that family history represents an important non-modifiable risk factor for conditions like hypertension and diabetes^62^, it becomes imperative to develop family-centered education initiatives and support networks aimed at fostering mutual support thereby improving compliance behavior.

From our qualitative analysis, it became apparent that *belief of consequences and emotions,* such as the fear of formal diagnosis and its associated healthcare cost, were important determinants of referral compliance. Although concerns about healthcare costs can be addressed through counseling of available subsidies and financial support, what has been deficient was addressing emotions, particularly when it is combined with *optimism,* such as low illness perceptions. This means that there is a scope to incorporate interventions targeting psychological barriers and enablers. Behavioral research suggests that over-emphasis on health risks triggers fear, while undermining risks increases blind optimism and diminishes motivation^63,64^. Therefore, a public health message on referrals should be framed to highlight the positive consequences, such as reassurance for negative results and potential positive health outcomes, and at the same time, raise awareness of anticipated regret if individuals opt not to adhere to the referral recommendations.

Another notable finding from interviews is related to the *external context and resources* that have implications for system-based improvements. Contrary to existing literature, our research shows that participants value reminders, facilitating compliance^65^. However, inadequate communication at the point of result collection appears to be one of the main factors influencing compliance. Hence, integrating “teachable moment” brief education and counseling at the point of result collection could be considered to increase the attention of the target audience and ensure an educated decision in the midst of uncertainty^66^. This brief education is most beneficial to those with limited *knowledge* and strong aversion to medications (*reinforcement*)*,* as identified by our study, and it should also address practical support to overcome unique barriers. Although knowledge alone may not result in behavior, it can inform beliefs, which in turn affect more proximal factors of behavior change like intentions and readiness, as shown in behavioral health theories^67^. Similarly, aversion to medications could be addressed by brief communication with individuals at the result collection, offering them a broader spectrum of choices to manage their health conditions while explaining the potential benefits of prescribed medicines. Optimization of primary care referral programs after screening is critical; as many non-compliant participants cited confusion related to scheduling an appointment with a primary care provider, prescheduling of appointments or an opt-out appointment system can be considered to improve compliance with recommendations as shown in other studies^68,69^. Healthier SG, a recent national initiative aimed at enrolling residents in their preferred family clinic for long-term personalized care, may offer a potential solution to the challenges associated with scheduling follow-up appointments^70^.

Our study has several strengths. Using both administrative data and theory-informed empirical interviews, the study comprehensively assessed the factors influencing non-compliance with physician referral recommendations. The TDF domains allowed us to systematically evaluate the determinants of behavior through a well-developed theoretical underpinning, which can facilitate evidence-based links to behavior change techniques. A recent systematic review of TDF applied literature highlighted the notable deficiency of non-Western studies, and therefore this study will make a meaningful contribution to the body of literature^71^. Limitations of this study include a skewed sample of the qualitative component with disproportionately more male and Chinese participants, and thus it is possible that we fail to capture distinct perspectives from female and other ethnic groups. Further research is warranted to understand the unique determinants among individuals from culturally diverse and minority backgrounds. Another potential limitation is that our qualitative data may not encompass all 14 domains of the TDF. However, we identified seven key domains as the most critical factors influencing non-compliance/compliance behavior through careful mapping of qualitative data. As this study was conducted in Singapore, an urban city-state, our findings may not be transferrable to rural and remote settings.

## Conclusions

Community health screenings serve as a vital initial step in detecting potential health issues, but their true impact hinges on individuals taking an initiative to follow through with physician referral recommendations based on their screening results. Our study shed light on key socioeconomic factors, including lower socioeconomic status, younger age, and Chinese ethnicity, which influence non-compliance behavior. At the same time, behavioral factors such as sense of personal control, awareness of health risks, optimistic outlook, social demands, fear of confirming a diagnosis, and systemic challenges play a pivotal role in shaping individuals’ decisions regarding non-compliance/compliance. Our mixed-methods approach had provided a complementary view of the key determinants and valuable implications for the development of interventions aimed at addressing modifiable factors and improving adherence to referral recommendations. Future endeavors should include targeted and culturally sensitive outreach efforts, evidence-based information dissemination, family-centered educational programs, framing public health messages to emphasize positive outcomes of actions, the incorporation of brief education and counseling during result collection and the implementation of an opt-out appointment system. These strategies could collectively contribute to a more effective approach to promoting follow-up care and ultimately improving health outcomes.

### Supplementary Information

Supplementary Information 1.

Supplementary Information 2.

## Data Availability

The datasets generated during the study are available from the corresponding author upon reasonable request.
