# Supplementary material for: Socioeconomic and behavioral determinants of non-compliance with physician referrals following community screening for diabetes, hypertension and hyperlipidemia: a mixed-methods study
Source: Sci Rep. 2023 Nov 23;13:20554. doi: 10.1038/s41598-023-47168-8 (PMC10667337; doi:10.1038/s41598-023-47168-8)
Supplement: Supplementary file 1 — Supplementary Information 1. [file 41598_2023_47168_MOESM1_ESM.pdf]

Semi-structured interview guide

Today, I would like to explore your experience and views of community health screening and follow-up procedures.

Experience of community-based chronic disease screening:

1. Tell me about your experience with respect to the community health screening program you have participated.
    a. What was your experience like when you participated in the screening?
    b. What motivated you to take part in the screening?

Perceived susceptibility and experience of self-management:

2. To what extent do you think you're aware of your condition? Can you elaborate?
3. If you have diabetes (high blood pressure), how do you think it would affect your life if left unattended?
4. What are some ways you have tried to change your lifestyle (diet, physical activity)? Tell me about things you tried but were unsuccessful.
5. How important is it for you to make lifestyle changes to reduce the risk of developing serious conditions such as heart attack or stroke? Why?

Result collection and referral recommendation:

6. Can you share with me about the result collection?
    a. Walk me through the process of how you received the screening results.
    b. How were the results handed to you? Who did you communicate with?
    c. What was your understanding of the results at that time?
    d. Did the staff at the result collection suggest you go and see a primary care doctor? If so, how did the staff explain to you?
    e. What was your initial reaction to the referral recommendation?

Nurse follow-up procedures:

7. I want to hear your views and experience regarding the telephone follow-up.
    a. Do you recall you received a telephone call from SingHealth nurses? If so, how many times did she contact you?
    b. What was the conversation between you and the nurse? What did the nurse tell you?
    c. How did you feel about the follow-up calls prompting you to schedule a doctor appointment?
    d. What was your reaction to the call?

Decision-making process:

8. How did you come to a decision to follow (or not to follow) the referral recommendation?
    a. Can you tell me what your initial decision was?
    b. How did the decision change over time?

Factors influencing decisions:

9. What factors influence your decision about the physician referral recommendation? Can you elaborate?
    Potential responses may be related to cost, competing priorities, perceived difficulty accessing GP Clinic, physical/health conditions, internal motivation, social influences, self-confidence, understanding of condition, optimism/pessimism, outcome expectancies, rewards, personal goals (Please do not use these as probes)

10. Which factors were the most important to you and why?

Implementation challenges and suggestions

11. How do you think the overall screening and referral recommendation can be improved to encourage people like yourself to comply with the recommendation?
    a. Are there any aspects of the programme that you think could have been better?
12. Is there anything else you would like to add on the topics we have discussed today?

Please do a brief recap of the main points. Ask if anything has been left out.

*Thanks for your participation. Your comments will be very helpful to us and the intervention we are planning for people with undiagnosed diabetes, hyperlipidaemia, and hypertension.
