# Supplementary material for: Socioeconomic and behavioral determinants of non-compliance with physician referrals following community screening for diabetes, hypertension and hyperlipidemia: a mixed-methods study
Source: Sci Rep. 2023 Nov 23;13:20554. doi: 10.1038/s41598-023-47168-8 (PMC10667337; doi:10.1038/s41598-023-47168-8)
Supplement: Supplementary file 2 — Supplementary Information 2. [file 41598_2023_47168_MOESM2_ESM.pptx]

## Slide 1
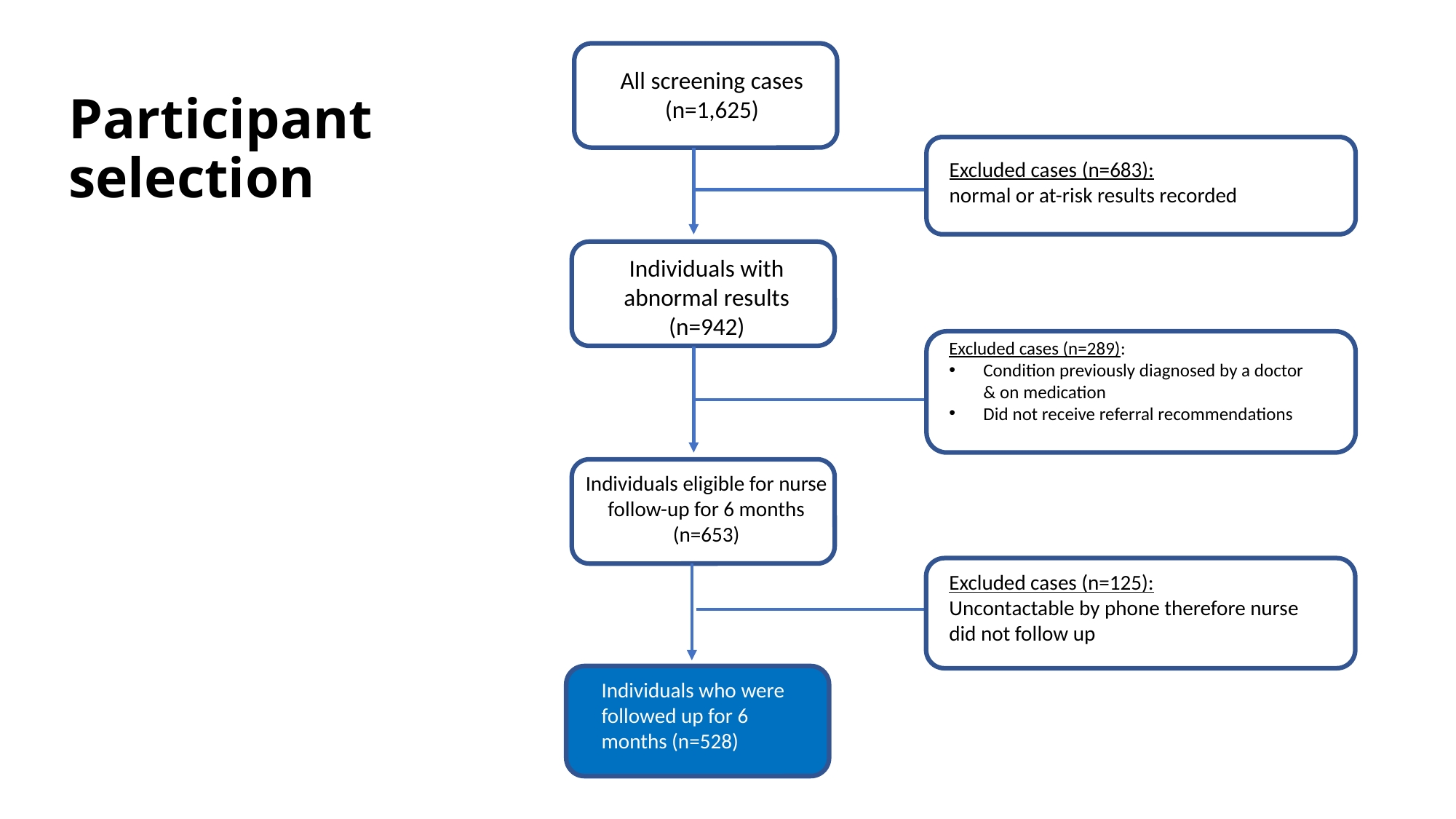

All screening cases (n=1,625)
# Participant selection
Ecl
Excluded cases (n=683):
normal or at-risk results recorded
i
Individuals with abnormal results (n=942)
Excluded cases (n=289):
Condition previously diagnosed by a doctor & on medication
Did not receive referral recommendations
iF
Individuals eligible for nurse follow-up for 6 months (n=653)
Excluded cases (n=125):
Uncontactable by phone therefore nurse did not follow up
Individuals who were followed up for 6 months (n=528)
